# Artificial intelligence-based radiomics for the prediction of nodal metastasis in early-stage lung cancer

**DOI:** 10.1038/s41598-023-28242-7

**Published:** 2023-01-19

**Authors:** Yoshihisa Shimada, Yujin Kudo, Sachio Maehara, Kentaro Fukuta, Ryuhei Masuno, Jinho Park, Norihiko Ikeda

**Affiliations:** 1 Department of Thoracic Surgery, Tokyo Medical University, 6-7-1 Nishishinjuku, Shinjuku-Ku, Tokyo, 160-0023 Japan; 2 Department of Radiology, Tokyo Medical University, Tokyo, Japan; 3 Department of Thoracic Surgery, Tokyo Medical University, Tokyo, Japan

**Keywords:** Non-small-cell lung cancer, Surgical oncology

## Abstract

We aimed to investigate the value of computed tomography (CT)-based radiomics with artificial intelligence (AI) in predicting pathological lymph node metastasis (pN) in patients with clinical stage 0–IA non-small cell lung cancer (*c*-stage 0–IA NSCLC). This study enrolled 720 patients who underwent complete surgical resection for *c*-stage 0–IA NSCLC, and were assigned to the derivation and validation cohorts. Using the AI software Beta Version (Fujifilm Corporation, Japan), 39 AI imaging factors, including 17 factors from the AI ground-glass nodule analysis and 22 radiomics features from nodule characterization analysis, were extracted to identify factors associated with pN. Multivariate analysis showed that clinical stage IA3 (*p* = 0.028), solid-part size (*p* < 0.001), and average solid CT value (*p* = 0.033) were independently associated with pN. The receiver operating characteristic analysis showed that the area under the curve and optimal cut-off values of the average solid CT value relevant to pN were 0.761 and -103 Hounsfield units, and the threshold provided sensitivity, specificity, and negative predictive values of 69%, 65%, and 94% in the entire cohort, respectively. Measuring the average solid-CT value of tumors for pN may have broad applications such as guiding individualized surgical approaches and postoperative treatment.

## Introduction

Pathological lymph node status is considered one of the most important prognostic factors in patients with early-stage non-small cell lung cancer (NSCLC)^1^. There is an approximately 15–20% risk of occult lymph node metastasis in patients with stage I disease^2–5^. The 5-year overall survival rates of patients with pathological N1, N2, and N3 NSCLC were 49%, 36%, and 20%, respectively^1^. Given that pathological lymph node metastasis (pN) is a substantial threat to survival, identifying the relevant clinical factors is highly beneficial when considering surgical approaches, the indication of adjuvant chemotherapies, optimal postoperative surveillance, and the prediction of prognostic outcomes in clinical stage IA NSCLC.

Various techniques, such as imaging and endoscopic modalities, have been reported to enable the stratification of patients with NSCLC according to their prognosis^6–8^. Our recent report showed that quantitative computed tomography (CT) histogram analysis of lung tumors obtained by extracting voxel values enables the non-invasive prediction of pN in patients with clinical stage 0–IA NSCLC^8^. Cho et al. showed that radiological pure solid tumor and solid-part size were associated with pN1 and N2 lymph node metastases in patients with stage I NSCLC^3^. Furthermore, we conducted an artificial intelligence (AI) analysis of three-dimensional (3D) lung tumor imaging, showing that solid-part volume calculated using AI software was associated with an unfavorable prognosis in patients with radiologically solid-predominant NSCLC^9^.

Radiomics is a high-throughput quantitative tool that converts medical images into a large amount of predefined computational data. The potential application of radiomics in predicting lymph node metastasis, treatment response, and clinical outcomes of patients with lung cancer has recently attracted much attention^10–14^. Radiomics-based approaches coupled with AI may serve as non-invasive and personalized decision support methods to identify prognostically high-risk cohorts with early-stage NSCLC. The purpose of our study was to evaluate CT-based radiomics analysis to preoperatively predict pN in patients with clinical stage 0–IA NSCLC.

## Patients and methods

### Patients

**Figure 1:**
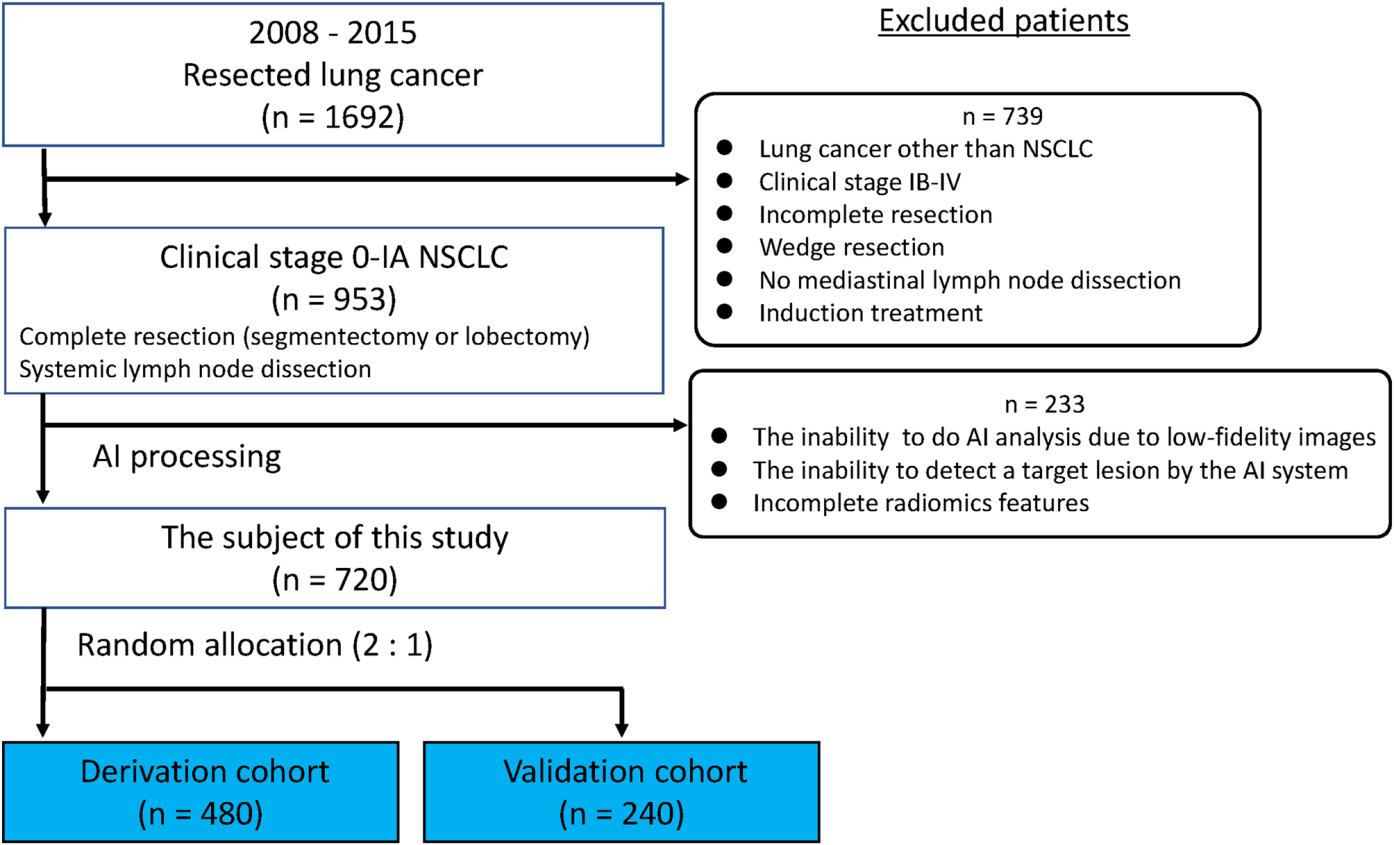
Consort diagram of patients included in the study. NSCLC—non-small cell lung cancer; ND—node dissection; AI—artificial intelligence.

There were 1692 patients who underwent pulmonary resection for lung cancer between January 2008 and December 2015. The following exclusion criteria were applied: lung cancer other than NSCLC, clinical stage IB–IV, incomplete surgical resection, wedge resection, no mediastinal lymph node dissection, and preoperative induction treatment. The remaining patients were those with clinical stage 0–IA NSCLC who underwent radical anatomical resection (lobectomy or segmentectomy) and systemic lymph node dissection at Tokyo Medical University Hospital. Among them, 233 patients were excluded because the AI imaging features of their lesions could not be processed. Low-fidelity CT images due to a limited number of CT slices and existing normal lung structures and non-malignant lesions resembling tumors cause the AI’s misrecognition to a target tumor. Finally, 720 patients for whom AI processing using their chest CT was successfully performed were enrolled in this study. We randomly assigned 480 and 240 patients to the derivation and validation sets to balance the proportions of patients with pN, respectively. A consort diagram of patients included in the study was shown in Fig. 1. We reviewed the medical records of each patient for preoperative clinical information including TNM stage. The TNM stage was determined according to the eighth edition of the TNM classification of malignant tumors. The comorbidities included diabetes mellitus, cardiovascular disease, chronic obstructive pulmonary disease, cerebral disease, autoimmune disease, interstitial pneumonia, and asthma.

### Patient follow-up

Patients were examined at the 6-month intervals for the first 2 years and 1-year intervals on an outpatient basis, with the aim of continuing follow-up for 10 years after resection. Follow-up evaluations included physical examinations, chest radiography, and blood tests. Chest and abdominal CT scans were performed every 6 months in the first 2 years and annually from the third year. Further evaluations, including brain magnetic resonance imaging and bone scintigraphy, were performed when the symptoms or signs of recurrence were observed. Positron emission tomography/computed tomography (PET/CT) was performed when appropriate. The date of recurrence was defined as the date of histologic proof or the date of identification based on clinicoradiologic findings by a physician.

### Radiological evaluation of primary tumor

All patients in this study underwent preoperative high-resolution CT and three-dimensional CT lung modeling. Helical CT images (1.25-mm-thick) were obtained from the whole lung. The whole tumor and solid-part sizes were preoperatively measured by two experienced thoracic radiologists (Dr. R.M. with 14 years and Dr. J.P. with 30 years of experience in chest CT interpretation, respectively). The solid-part size was defined as the maximum diameter of the solid component of the lung window, excluding the ground-glass nodules.

### Radiomics and AI imaging analysis of CT images

After the lung CT Digital Imaging and Communications in Medicine (DICOM) format data were transmitted to the Synapse Vincent system (Fujifilm Corporation, Tokyo, Japan), the AI software Beta Version (AI software; Fujifilm Corporation) in the system automatically detected and segmented lung nodules in the bilateral lungs and reconstructed the 3D images of the lungs and nodules. This segmentation algorithm was based on the 3D-Convolutional Neural Network using a modified *U*-Net architecture. The network consisted of 17 convolutional layers. The system separated the solid-part of lung nodules from non-solid-parts (ground-glass nodules; [GGNs]), and determined the size, volume, and ratio of solid-part, non-solid-part, and whole tumor lesions, as well as CT histogram features. The total of 39 AI imaging features included 22 radiomics features and 17 features from the GGN analysis (Supplemental Table 1), and the radiomics features were automatically extracted and displayed as a score from 0 to 1 using a feature analysis function. The 22 radiomics features were based on the labeling of 5118 tumors, and the datasets of the developmental process were divided into training, validation, and test sets. The trained model gave a mean area under the curve (AUC) score of 0.93 for all features on the test dataset based on the information of radiological and histological diagnoses of tumor and non-tumor lesions (data not shown). This AI lung nodule analysis model uses a convolutional neural network based on VGG-16 and consists of 12 layers of convolution, with four layers removed from the output side of the VGG-16. To extract 3D radiomics features, 3D convolution was used for all the convolution layers. The 3D software automatically generated the 17 features from the GGN analysis. The software determined the volumes of the GGNs, radiologically solid lesions and whole tumor lesions, the ratios of GGNs or the radiologically solid lesions, and CT histograms data by the feature analysis function.

**Table 1:** Patient characteristics.

Variable	Derivation cohort *n* = 480 (%)	Validation cohort *n* = 240 (%)	*p* value
Age, years (mean ± SD)	23–86 (66 ± 10)	38–87 (67 ± 10)	0.245
Sex, male	236 (49)	122 (51)	0.673
Any smoking history	271 (57)	146 (61)	0.262
Comorbidities, present	227 (47)	132 (55)	0.051
FEV_1.0_, L (mean ± SD)	0.98–4.93 (2.36 ± 0.67)	0.89–4.93 (2.28 ± 0.64)	0.125
FEV_1.0_% (mean ± SD)	39–96 (73 ± 9)	35–93 (73 ± 10)	0.795
Clinical stage			(0-IA2 vs. IA3) 0.163
0	32 (7)	13 (6)	
IA1	94 (20)	43 (18)	
IA2	201 (42)	95 (40)	
IA3	153 (32)	89 (37)	
Pathological stage			(I vs. II-III) 0.234
IA	312 (65)	164 (68)	
IB	92 (19)	46 (19)	
II	40 (8)	16 (7)	
III	36 (8)	14 (6)	
Histology			0.274
Adenocarcinoma	422 (88)	204 (85)	
Others	58 (12)	36 (15)	
Pathological lymph-node status			(N0 vs. N1-3) 0.869
N0	420 (88)	213 (89)	
N1	25 (5)	13 (5)	
N2	31 (7)	14 (6)	
N3	4 (1)	0	
Surgical procedure			0.008
Lobectomy	430 (90)	229 (95)	
Segmentectomy	50 (10)	11 (5)	

*SD* standard deviation; *FEV*forced expiratory volume.

### Statistical analysis

Overall survival (OS) was measured from the day of surgery to the day of death from any cause or the day on which the patient was last known to be alive. Recurrence-free survival (RFS) was measured as the interval between the date of surgery and date of recurrence, date of death from any cause, or date the patient was last known to be alive. OS and RFS curves were plotted using the Kaplan–Meier method, and differences in variables were determined using the log-rank test. Univariate and multivariate logistic regression analyses were performed to identify the factors associated with pN using a Cox proportional hazards model. A backward stepwise selection method was used to build logistic regression models, and variables with a threshold of *p* < 0.15 were adopted for the stepwise model selection procedure to prevent overlooking relevant factors. We conducted univariate and multivariate analyses separately using the 39 AI imaging features and other clinical factors. Pearson’s chi-square test (for categorical data) and Student’s *t* test (for continuous data) were used to compare two groups of data. Receiver operating characteristics (ROC) curves for lymph node metastasis and early recurrence were constructed, and the optimal cut-off values were determined using the AUC. All tests were two-sided, and statistical significance was set at *p* < 0.05. The SPSS statistical software package (version 28.0, DDR3 RDIMM; SPSS Inc., Chicago, IL, USA) was used for statistical analysis. Violin plots were constructed using the R package (version 4.0.5).

### Ethical statement

The authors are accountable for all aspects of the work in ensuring that questions related to the accuracy or integrity of any part of the work are appropriately investigated and resolved. All procedures performed in this study involving human participants were performed in accordance with the Declaration of Helsinki (as reserved in 2013). The study was approved by the institutional review board of Tokyo Medical University (SH3951). Informed consent for the use and analysis of clinical data was obtained preoperatively for each patient.

## Results

Patient characteristics are shown in Table 1. The derivation cohort included 236 men (49%) and 244 women (51%), while the validation cohort included 122 men (51%) and 118 women (49%). There were no significant differences between the two cohorts, except for surgical procedures. Segmentectomy was performed in 50 patients (10%) in the derivation cohort and in 11 patients (5%) in the validation cohort (*p* = 0.008).

**Figure 2:**
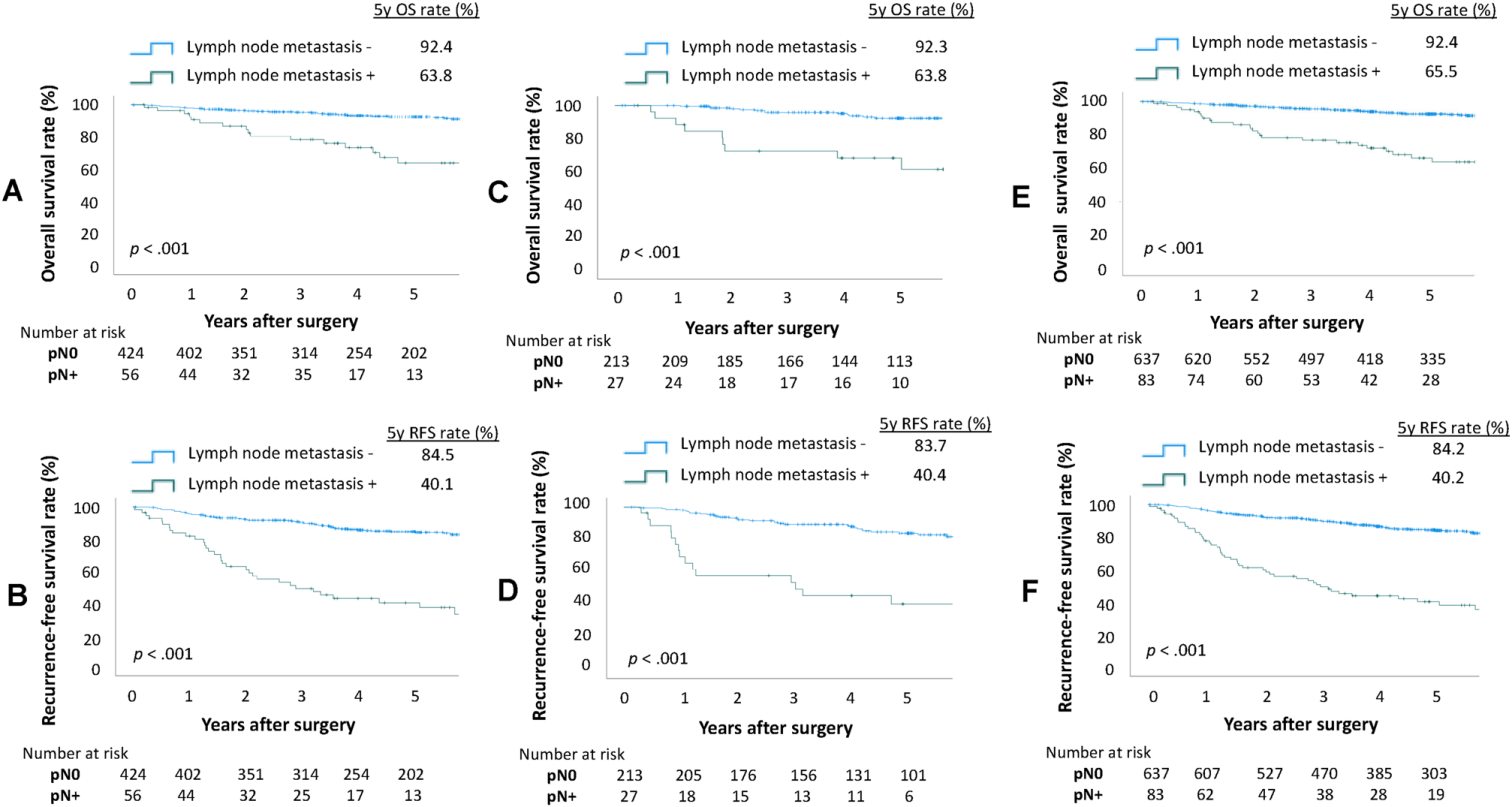
Overall survival and recurrence-free survival of clinical stage 0–IA patients according to status of lymph node metastasis status. (**A**) Overall survival and (**B**) recurrence-free survival of patients in the derivation cohort. (**C**) Overall survival and (**D**) recurrence-free survival of patients in the validation cohort. (**E**) Overall survival and (**F**) recurrence-free survival of patients in the entire cohort. pN0—pathological lymph node negative; pN + —pathological lymph node positive.

Fifty-six patients (12%) in the deviation cohort and 27 patients (11%) in the validation cohort were found to have positive lymph nodes. Kaplan–Meier curves showed that pN status was significantly associated with OS and RFS in the derivation cohort (5-year OS rate 92.4% vs. 63.8%, *p* < 0.001, Fig. 2A; and 5-year RFS rate 84.5% vs. 40.1%, *p* < 0.001, Fig. 2B), the validation cohort (5-year OS rate 92.3% vs. 63.8%, *p* < 0.001, Fig. 2C; and 5-year RFS rate 83.7% vs. 40.4%, *p* < 0.001, Fig. 2D), and in the entire cohort (5-year OS rate 92.4% vs. 65.5%, *p* < 0.001, Fig. 2E; and 5-year RFS rate 84.2% vs. 40.2%, *p* < 0.001, Fig. 2F).

**Figure 3:**
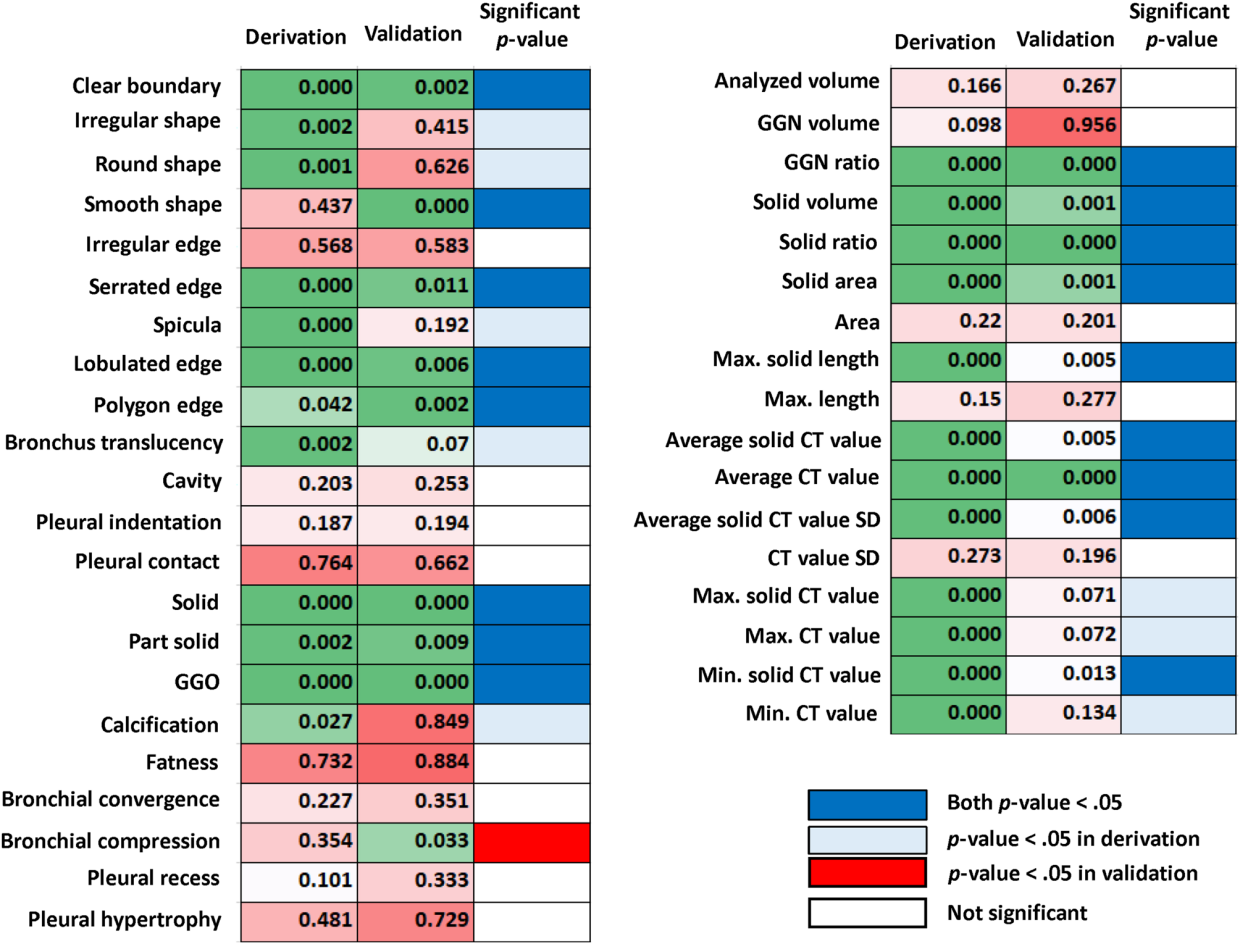
Heat map showing the *p*-value significance of correlations between the 39-feature signatures and pathological lymph node metastasis. Artificial intelligence imaging analysis indicates the signatures related to lymph node metastasis in the derivation and validation cohorts.

The *p*-value significance of the correlations between the 39 AI imaging features and the pN is shown in Fig. 3, and 17 factors were found to be significant in both cohorts. Univariate analyses of RFS in the derivation using clinical factors such as age, sex, smoking habit (presence versus absence), FEV1.0%, comorbidities (presence versus absence), solid-part size, clinical stage (0–IA2 versus IA3), and surgical procedure (lobectomy versus segmentectomy) showed that larger solid-part size (hazard ratio [HR] 2.82, 95% confidence interval (CI) 1.98–4.02, *p* < 0.001) and clinical stage IA3 (HR 2.36, 95%CI 1.49–3.76, *p* < 0.001) were found to be significant unfavorable RFS factors (Table 2). Solid-part size (HR 7.96, 95%CI 3.26–19.48, *p* < 0.001) and clinical stage IA3 (HR 3.23, 95%CI 1.13–9.19, *p* = 0.028) were also independently associated with poor RFS on multivariate analysis (Table 2). Among the AI imaging features, the average solid CT value (HR 1.01, 95%CI 1.00–1.02, *p* = 0.033) was the only independent factor associated with unfavorable RFS on multivariate analysis (Table 2).

**Table 2:** Univariate and multivariate analyses of lymph node metastasis in the derivation cohort.

Factors among clinicopathological factors	Univariate analysis	
	Hazard ratio (95% CI)	*p* value
Age	1.02 (1.00–1.04)	0.086
Sex (male vs. female)	1.45 (0.91–2.30)	0.118
Smoking	1.20 (0.78–1.86)	0.407
FEV_1.0_%	0.77 (0.55–1.07)	0.122
Comorbidities	1.36 (0.86–2.16)	0.191
Solid-part size	2.82 (1.98–4.02)	< 0.001
Clinical stage (IA3 vs. 0-IA2)	2.36 (1.49–3.76)	< 0 .001
Procedure (lobectomy vs. segmentectomy)	2.67 (0.82–8.73)	0.104
Factors among clinicopathological factors	Multivariate analysis	
	Hazard ratio (95% CI)	*p* value
Solid-part size	7.96 (3.26–19.48)	< 0.001
Clinical stage (IA3 vs. 0-IA2)	3.23 (1.13–9.19)	0.028
Procedure (lobectomy vs. segmentectomy)	3.34 (0.43–25.65)	0.247
Factors among AI imaging features	Multivariate analysis	
	Hazard ratio (95% CI)	*p* value
Average solid CT value	1.01 (1.00–1.02)	0.033
Maximum solid CT value	1.00 (1.00–1.01)	0.084
Bronchus translucency	3.10 (0.80–1.90)	0.101

*CI* confidence interval; *FEV* forced expiratory volume; *AI* artificial intelligence; *CT* computed tomography.

To investigate the effect of the statistically significant predictive factors, solid-part size, and average solid CT value on pN, we calculated ROC curves in the derivation cohort (Supplementary Figure. 1). The AUC and optimal cut-off values relevant to pN were 0.754 and 1.83 cm for solid-part size and 0.761 and − 103 Hounsfield units (HU) for the average solid CT value.

**Figure 4:**
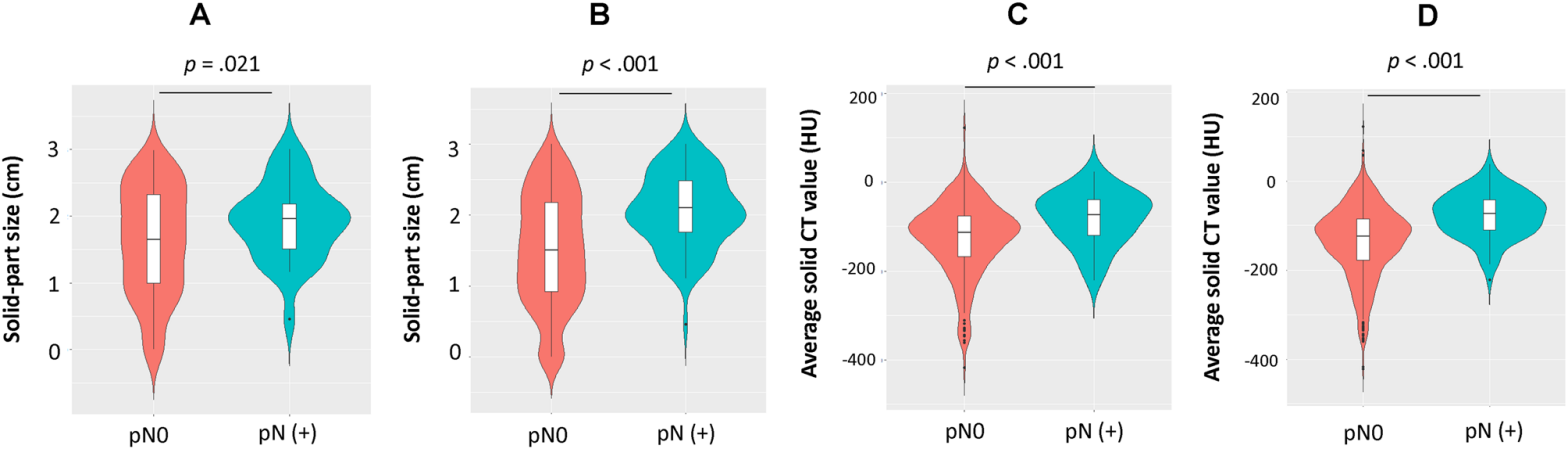
Violin plots for the comparison of the distribution of solid-part size in the validation cohort (**A**) and entire cohort (**B**), and average solid CT value in the artificial intelligence in the validation cohort (**C**) and entire cohort (**D**). pN( +)—pathological lymph node positive; CT—computed tomography; HU—Hounsfield units.

Violin plots were constructed to visualize the comparative distributions of the solid-part size of the validation (Fig. 4A) and the entire cohort (Fig. 4B), and the average solid CT value of the validation (Fig. 4C) and the entire cohort (Fig. 4D) to analyze the association with pN. Significant differences were observed in the solid-part size in the validation (*p* = 0.021) and the entire cohort (*p* < 0.001), and in the average solid CT value in the validation (*p* < 0.001) and entire cohort (*p* < 0.001). Patients in the derivation, validation, and entire cohorts were dichotomized at 1.83 cm of solid-part size, which showed pN ratios of 23% and 4% (*p* < 0.001), 15% and 8% (*p* = 0.114), and 20% and 6% (*p* < 0.001) for the high- and low-risk cohorts, respectively. Those in the derivation, validation and entire cohorts were dichotomized at − 103 HU of the average solid CT value, showing a pN ratio of 22% and 6% (*p* < 0.001), 17% and 7% (*p* = 0.011), and 20% and 6% (*p* < 0.001) for the high and low-risk cohorts, respectively (Table 3). The threshold provided sensitivities of 70%, 67%, and 69%, specificities of 68%, 59%, and 65%, and negative predictive values of 94%, 93%, and 94% in the derivation, validation, and entire cohorts, respectively (Table 3).

**Table 3:** Correlation between the significant values and lymph node metastasis.

Cohort	Solid-part size	pN (%)	*p* value	Sensitivity (%)	Specificity (%)	Accuracy (%)	NPV (%)
Derivation	> 1.83 cm	44 (23)	< 0.001	44/56 (79)	278/424 (66)	322/480 (67)	278/290 (96)
	< 1.83 cm	12 (4)					
Validation	> 1.83 cm	16 (15)	0.114	16/27 (59)	121/213 (57)	137/240 (57)	121/132 (92)
	< 1.83 cm	11 (8)					
All	> 1.83 cm	60 (20)	< 0 .001	60/83 (72)	399/637 (63)	459/720 (64)	399/422 (95)
	< 1.83 cm	23 (6)					
Cohort	Average solid CT value	pN (%)	*p* value	Sensitivity (%)	Specificity (%)	Accuracy (%)	NPV (%)
Derivation	> − 103 HU	39 (22)	< 0.001	39/56 (70)	288/424 (68)	327/480 (68)	288/305 (94)
	< − 103 HU	17 (6)					
Validation	> − 103 HU	18 (17)	0.011	18/27 (67)	126/213 (59)	144/240 (60)	126/135 (93)
	< − 103 HU	9 (7)					
All	> − 103 HU	57 (20)	< 0 .001	57/83 (69)	414/637 (65)	471/720 (65)	414/440 (94)
	< − 103 HU	26 (6)					

*pN* pathological lymph node metastasis; *NPV* negative predictive value; *CT* computed tomography; *HU* Hounsfield units.

## Discussion

In the present study, we found that the average solid-CT value of the tumor extracted from the AI imaging analysis as well as the clinical stage and the solid-part size of the tumor, were independently associated with pN in patients with clinical stage 0–IA NSCLC. The AUC of the average solid CT value for pN was 0.761, and the cut-off level was − 103 HU, which is a better threshold associated with pN than a solid-part size on chest CT based on the results of the pN ratio and accuracy.

Preoperative identification of the boundary between high- and low-risk populations on the pN is crucial for optimal surgical procedures, particularly in clinical stage I NSCLC^3,5,8,15^. Cho et al. showed that higher clinical stage and larger solid tumor size were associated with pN, consistent with our results^3^. Koike et al. identified four significant predictive factors including CTR for pN in patients with stage IA NSCLC^4^. Their patients were dichotomized at 0.89 of CTR, which showed a sensitivity of 98%, specificity of 43% and accuracy of 47%, respectively^4^. Kaseda et al. demonstrated that the SUVmax of the tumor was independently associated with occult lymph node metastasis in patients with stage I NSCLC, and the thresholds of the SUVmax provided a sensitivity of 68%, specificity of 53% and accuracy of 55%, respectively^15^. Even though those studies showed higher sensitivities for pN, lower specificities ultimately led to lower accuracies than our results. We recently showed that quantitative CT histogram analysis of lung tumors contributed to the noninvasive prediction of pN in patients with clinical stage IA NSCLC^8^. Average solid CT value obtained in the current study is one of the CT histogram parameters, and growing evidence suggests that quantitative CT histogram analysis of lung cancer is helpful in detecting imaging variables associated with postoperative outcomes and histologic invasiveness^16–19^. Numerous other studies have reported factors associated with pN, such as age, histology, maximum standardized uptake values on PET/CT, solid-part size, and the maximum consolidation diameter to the maximum tumor diameter (CTR) in early-stage NSCLC^20–23^. The ratio of CTR and solid-part size seen on HRCT has a greater chance of identified a pathological invasive component^21,24^. Patients with a larger proportion of solid-part size had a higher recurrence rate regardless of surgical procedures^24,25^. The present study demonstrated that the solid-part size was significantly associated with pN. HRCT plays a vital role in the diagnosis, and clinical decision making, and predicting patient outcomes in early-stage NSCLC. However, it is important to note that lung lesions with an irregularly shaped solid-part in the greatest dimension sometimes cause inter- and intra-observer variability.

Several studies have reported that radiomics approaches were highly useful for predicting pN in patients with NSCLC^13,26,27^. Cong et al. established a radiomics model in predicting pN in early-stage NSCLC, and the predictive performance of their radiomics model was significantly better than that of clinical factor-based model^13^. Radiomics implementation comprises several processes, such as imaging, feature extraction, feature selection, signature building, and analysis. Usually, the region of interest on CT images is manually outlined by experienced radiologists. By contrast, the current radiomics analysis demonstrated that 39 AI imaging features could be automatically extracted and displayed as measures and scores. We believe that our work may serve as a promising method for predicting pN in a non-invasive manner, allowing physicians without technical expertise in the context of image synthesis to easily conduct AI analysis to unravel tumor phenotypic characteristics.

This study has several limitations. First, it was a retrospective review of patients from a single institution, and inherent biases existed. Second, not all preoperative CT images from patients were successfully processed using our radiomics analysis. There were cases in which a radiomics signature could not be obtained because of low-fidelity CT images and target lesions unrecognized by the AI system. Third, our study only used CT imaging features. PET/CT is highly useful for predicting prognosis and detecting pathological invasive factors, including lymph node metastasis, even though there are various causes of false-positive and false-negative results^6,23^. However, 49% of patients were assessed using the same PET/CT setting, whereas the remaining were assessed by other scanners or had no PET/CT examinations in this study (data not shown). Therefore, we excluded data derived from PET/CT from the analyses. Fourth, the AI used in this study was specific to the software package used by our group. Applying these results to other centers would require the use of the same software. Therefore, external validation of this study outside our center could not be performed currently. Finally, the cut-off values from the ROC curves, such as − 103 HU on the average solid CT value and 1.83 cm on the solid-part size for pN were arbitrary. Hence, the results can vary depending on the number of patients or type of CT scanning protocol used although we performed the validation analyses to see if these thresholds were useful to predict pN.

In conclusion, AI software in CT-based radiomics provides significant imaging features for the prediction of pN in patients with clinical stage 0–IA NSCLC. Measuring the average solid CT value of tumors for pN may have broad clinical applications such as guiding surgical approaches and individualized postoperative treatment.

## Supplementary Information

Supplementary Information 1.

Supplementary Information 2.

## Data Availability

The analyzed data in this article will be shared on reasonable request to the corresponding author, except for the deviation and validation dataset for the radiomics analysis by AI software.

## Abbreviations

AI: Artificial intelligence
AUC: Area under the curve
CI: Confidence interval
CT: Computed tomography
GGN: Ground-glass nodule
HR: Hazard ratio
HU: Hounsfield units
NPV: Negative predictive value
NSCLC: Non-small cell lung cancer
OS: Overall survival
PET: Positron emission tomography
RFS: Recurrence-free survival
ROC: Receiver-operating characteristics
SD: Standard deviation
3D: Three-dimensional
