# Supplementary material for: Artificial intelligence-based radiomics for the prediction of nodal metastasis in early-stage lung cancer
Source: Sci Rep. 2023 Jan 19;13:1028. doi: 10.1038/s41598-023-28242-7 (PMC9852472; doi:10.1038/s41598-023-28242-7)
Supplement: Supplementary file 1 — Supplementary Information 1. [file 41598_2023_28242_MOESM1_ESM.pptx]

## Slide 1
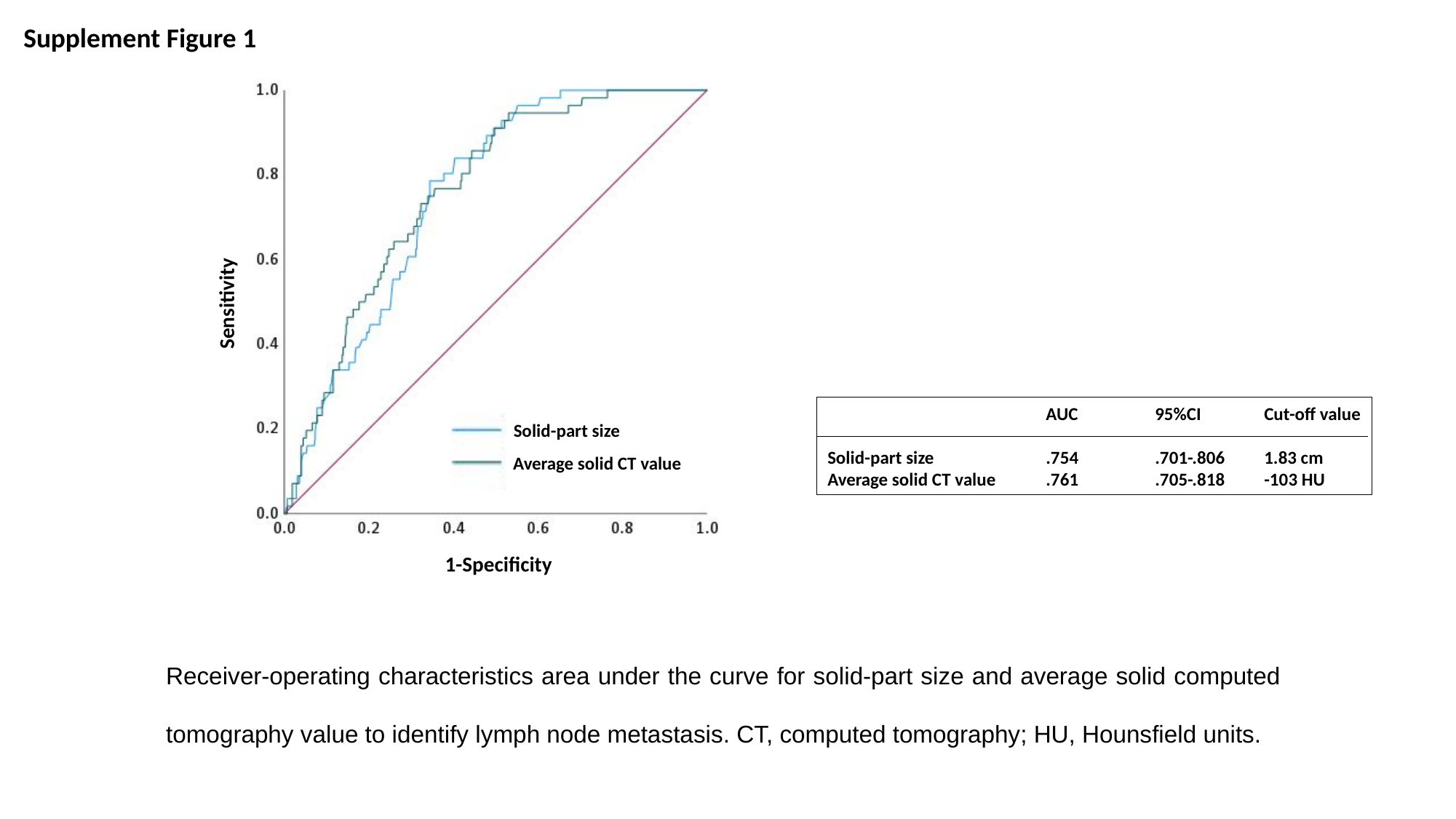

Supplement Figure 1
Sensitivity
		AUC	95%CI	Cut-off value
Solid-part size		.754	.701-.806	1.83 cm
Average solid CT value	.761	.705-.818	-103 HU
Solid-part size
Average solid CT value
1-Specificity
Receiver-operating characteristics area under the curve for solid-part size and average solid computed tomography value to identify lymph node metastasis. CT, computed tomography; HU, Hounsfield units.
