# Supplementary material for: Artificial intelligence-based radiomics for the prediction of nodal metastasis in early-stage lung cancer
Source: Sci Rep. 2023 Jan 19;13:1028. doi: 10.1038/s41598-023-28242-7 (PMC9852472; doi:10.1038/s41598-023-28242-7)
Supplement: Supplementary file 2 — Supplementary Information 2. [file 41598_2023_28242_MOESM2_ESM.docx]

***Supplemental Table 1.*** **List of radiomic features extracted using feature analysis model**

| Category | Class | Details |
| --- | --- | --- |
| Shape | Irregular shape | The quality of not being regular in nodular shape |
|  | Round shape | The quality of how closely the shape approaches that of a circle |
|  | Smooth shape | A texture without roughness |
| Margin | Clear boundary | The degree of clearness in nodular edge |
|  | Irregular edge | The degree of irregularity in nodular edge |
|  | Serrated edge | Having a notched edge or sawlike teeth in nodular edge |
|  | Spicula | A small needlelike structure in nodular edge |
|  | Lobulated edge | The quality made up of or having lobules in nodular edge |
|  | Polygon edge | The quality of a flat shape with three or more straight sides in nodular edge |
| Internal characteristics | Bronchus translucency | Air-filled bronchi being made visible by the opacification of surrounding alveoli |
|  | Cavity | An abnormal, thick-walled, air-filled space within a nodule |
|  | Calcification | Calcification within a nodule |
|  | Fatness | Fat within a nodule |
| External characteristics | Pleural contact | A nodule with pleural contact |
|  | Pleural indentation | A nodular indentation of the visceral pleura |
|  | Bronchial convergence | Bronchus converging to a nodule without adjoining or contacting the edge of the nodule |
|  | Bronchial compression | Bronchus compressing a nodule without adjoining or contacting the edge of the nodule |
|  | Pleural recess | Retraction of the pleura toward a nodule with a linear structure |
|  | Pleural hypertrophy | A state that causes thickening of the lung lining or pleura |
| Opacity | Solid | A nodule that completely obscures the entire lung parenchyma within it |
|  | Part solid | A nodule predominantly containing solid component with GGO |
|  | GGO | A nodule predominantly containing an area of hazy opacification or increased attenuation |
| Features from the GGN analysis | Analyzed volume | The whole tumor volume |
|  | GGN volume | The GGN-part volume |
|  | GGN ratio | The ratio of the GGN-part volume |
|  | Solid volume | The solid-part volume |
|  | Solid ratio | The ratio of the solid-part volume |
|  | Solid area | The maximum dimension of the solid-part of the lung windows excluding non-solid-part |
|  | Area | The maximum dimension of the whole tumor area |
|  | Maximum solid length | The maximum diameter of the solid-part of the lung windows excluding non-solid-part |
|  | Maximum length | The maximum diameter of the tumor |
|  | Average solid CT value | A CT histogram parameter measuring 50 percentile CT attenuation value of the sold-part |
|  | Average CT value | A CT histogram parameter measuring 50 percentile CT attenuation value of the tumor |
|  | Average solid CT value SD | SD of a CT histogram parameter measuring 50 percentile CT attenuation value of the sold-part |
|  | Average CT value SD | SD of a CT histogram parameter measuring 50 percentile CT attenuation value of the tumor |
|  | Maximum solid CT value | A CT histogram parameter measuring maximum CT attenuation value of the sold-part |
|  | Maximum CT value | A CT histogram parameter measuring maximum CT attenuation value of the tumor |
|  | Minimum solid CT value | A CT histogram parameter measuring minimum CT attenuation value of the sold-part |
|  | Minimum CT value | A CT histogram parameter measuring minimum CT attenuation value of the tumor |

GGO = ground-glass opacity, GGN = ground-glass nodule, CT = computed tomography, SD = standard deviation.
